# Can Consultation by a Clinical Pharmacist Prevent Morbidity and Mortality in Patients Undergoing Bariatric Surgery?

**DOI:** 10.3390/jcm13020310

**Published:** 2024-01-05

**Authors:** Carmil Azran, Almog Eliyahu Dahan, Orly Shimoni, Dror Dicker, Ariel Hammerman, Arik Dahan

**Affiliations:** 1 Department of Medical Technologies, Maccabi Healthcare Services, Tel-Aviv 6772168, Israel; 2 School of Pharmacy, Faculty of Medicine, The Hebrew University, Jerusalem 9112102, Israel; 3 Department of Clinical Pharmacology, School of Pharmacy, Faculty of Health Sciences, Ben-Gurion University of the Negev, Beer-Sheva 8410501, Israel; almogdah@post.bgu.ac.il (A.E.D.); orlyr@clalit.org.il (O.S.); 4 Internal Medicine Department, Hasharon Hospital-Rabin Medical Center, Petah Tikva 4937211, Israel; drord@clalit.org.il; 5 Faculty of Medicine, Tel Aviv University, Tel-Aviv 6997801, Israel; 6 Clalit Health Services, Tel-Aviv 6209804, Israel; arielha@clalit.org.il

**Keywords:** bariatric surgery, clinical pharmacist, medication management, complications, readmission, pharmacotherapy

## Abstract

The purpose of this work was to investigate the effect of clinical pharmacist consultation on the long-term morbidity and mortality outcomes among patients undergoing bariatric surgery. In this retrospective cohort study, 165 bariatric patients at Herzliya Medical Center who were identified as complex cases and were consulted by a clinical pharmacist (2013–2019) were compared with a wider group of bariatric patients with chronic diseases who were recorded in the Israeli General Bariatric Registry during the same years. The primary outcomes were rates of surgical complications, re-hospitalizations, and death up to one year after surgery. The secondary outcome was the rate of re-hospitalizations in different time periods. The twelve (12)-month rate of re-hospitalization in the intervention group was 10.9% vs. 19.5% in the comparison group (*p* = 0.005); the rate of documented postoperative complications was 2.7% vs. 3.9% (*p* = 0.462), and mortality was null vs. 0.16%, respectively. As for the secondary outcomes, the rates of re-hospitalizations in the periods of 0–30, 31–90, 91–180, and 181–365 days after surgery were 1.8% vs. 5.3% (*p* = 0.046), 2.4% vs. 4.1% (*p* = 0.278), 3.6% vs. 4.8% (*p* = 0.476), and 7.3% vs. 9.9% (*p* = 0.256) in the intervention vs. comparison cohorts, respectively. In conclusion, this study demonstrates the importance and benefit of referring to a specialized clinical pharmacist around bariatric surgery for improving patient safety, especially in complex patients. This is the first study to look at the long-term effects of clinical pharmacist consultation on re-hospitalization and mortality among bariatric patients, and our encouraging outcomes should hopefully stimulate more studies to show the invaluable role of specialized clinical pharmacists.

## 1. Introduction

In recent decades, there has been a constant increase in the rate of morbid obesity. Excess weight has been defined by the World Health Organization as a worldwide epidemic. This increase is recorded not only in adults but also in children and adolescents, and there is growing concern about the continuation of the upward trend in the coming years [1].

Obesity is defined by a body mass index (BMI) of 30 kg/m^2^ or higher. Severe obesity is usually defined as a BMI of 40 kg/m^2^ or higher [2]. According to the Organization for Economic Co-operation and Development (OECD) data, more than half of the population is overweight, one in four people is obese, and the rate of morbid obesity is increasing at a significant rate [3]. People with obesity are at an increased risk of morbidities, which include type 2 diabetes, hypertension, blood lipid disorders, cardiovascular disease, fatty liver, infertility, breathing problems, depression, anxiety, and even an increased risk of certain types of cancer [4,5].

Over the years, to help deal with the increasing rise in obesity and its consequences, several treatment approaches have been developed, including lifestyle interventions, drug therapy, and bariatric surgery. However, behavioral changes and drug treatment have been reported to have limited, temporary, and short-term effectiveness [6,7]. In addition, drug treatments for weight loss are sometimes accompanied by side effects, interactions between drugs, and financial burdens, which limit their use in the general patient population [8]. For these reasons, bariatric surgery has gained major momentum and has become the most effective solution known to date for those suffering from morbid obesity, both for weight loss and improving comorbidities [9,10].

However, bariatric surgeries may also have complications in the period close to the operation as well as later [11]. The early complications include leakage, bleeding, thromboembolic events, infections, pulmonary complications, and even death. Later complications include stenosis in the launch area, ulcers, reflux, nutritional deficiencies, and changes in bone mass [12]. Other side effects include multiple vomiting episodes and an increased risk of dehydration; abnormal laboratory indicators, including changes in blood sugar levels; abnormal INR levels; toxic levels of drugs in the blood; and others [13].

In addition, bariatric surgery is accompanied by anatomical changes that may affect the pharmacokinetic profile and the pharmacodynamics of many drugs. These changes include, among others, a decrease in stomach acidity [14], a decrease in the stomach volume, and bypass of the proximal part of the intestine and thus a decrease in surface area for absorption, and various enzymes and transporters that take part in drug metabolism and distribution [15,16,17]. Also, many changes may be required in the patient’s chronic medication dosing due to an improvement in chronic diseases such as diabetes, hypertension, and hyperlipidemia. Other medications may require a new titration, such as lithium and anti-epileptic medicines. These changes are an extreme challenge in the management of drug treatment in patients after bariatric surgery [18,19].

To date, there is a scarcity of scientific literature regarding the outcomes of clinical pharmacist consultation in patients undergoing bariatric surgery. Therefore, we sought to examine the effect of dedicated drug counseling provided by a clinical pharmacist on the rate of complications, re-hospitalizations, and mortality among patients who had undergone bariatric surgery.

## 2. Methods

We performed a retrospective cohort study in which we compared the results of bariatric patients who received counseling from a clinical pharmacist specializing in the pharmacology of bariatric surgery in the years 2013 to 2019 using the results of a wide cohort of patients with chronic diseases who underwent bariatric surgery and were recorded in the Israeli bariatric registry in the same years.

Consultations were performed mostly before surgery or during hospitalization after surgery as a preparation for the patients; some of the consultations were requested by the surgeons with a question of selecting the safest surgery for the patient with consideration of their medications and comorbidities. During the consultation, the clinical pharmacist performed medication and supplement reconciliation, examined the patients’ past medical history, and evaluated the blood laboratory results. Consultations included recommendations for formulation changes of chronic medications to a form that could be crushed or a liquid form; laboratory tests that should be performed before and after surgery, such as medication blood levels, when relevant; and chronic condition monitoring, for example, in diabetes, hypertension, hyperthyroidism, etc. Patients were taught how to self-monitor their diabetes, hypertension, and mental health and were informed about when they should turn to their primary care provider and ask for therapy evaluation, such as in cases of low blood pressure or low blood glucose. Other recommendations included the discontinuation of medications that are not advisable after surgery and alternative therapies, such as those causing hypoglycemia, diuretics, non-steroidal anti-inflammatory drugs (NSAIDs), oral birth control medications, etc. [4,19]. Supplements were also recommended, and their lifelong use was strongly advised. Many of the patients were taking psychiatric medications at the time of the consultation and were advised on self-monitoring and how absorption might be affected, and they received a treatment plan. Recommendations on medications were also written and discussed with the hospital staff, ambulatory-care providers, and surgeons. By the end of the consultation, patients received a written letter with all the recommendations, and this was also kept in their hospital medication records for other healthcare providers to see.

The Israeli National Registry for Bariatric Surgery was established in 2013 to gather data on all bariatric surgeries performed in Israel. The registry includes data on bariatric patients prior to their operation; data from the hospital performing the surgery; reports by health maintenance organizations (HMOs) after operation; information from questionnaires that are sent to all patients undergoing bariatric surgery; and data gathered by the pre-surgery bariatric committee, consisting of a physician, a dietitian, and a psychologist or social worker who approved the surgery on the basis of the patient’s comorbidities and psychological status [20].

The study intervention population included all patients, members of Clalit Health Services (CHS), who underwent operations at Herzliya Medical Center (HMC) and were identified by the treating staff as complex cases requiring drug counseling. Complex patients included patients with chronic diseases (medically treated diabetes, neurological conditions, psychiatric conditions, and more), medications requiring special monitoring, and cases in which surgeons consulted with a clinical pharmacist in order to choose the type of bariatric surgery on the basis of the patient’s medication. Herzliya Medical Center is a private hospital specializing in surgeries and has a unique bariatric committee that meets every patient before surgery. CHS is a large healthcare organization that covers about 52% of the entire Israeli population.

Patients undergoing bariatric surgery have appointments with the clinical pharmacist at HMC. On average, there is one meeting for each patient lasting between 30 and 60 min. During these consultations, clinical recommendations are provided to the patients.

Clinical pharmacist consultations include, among other things, the discontinuation of medications before surgery (such as NSAIDS, sulfonylureas, and diuretics), adjustments to medication dosage (insulin units and antihypertensive dosage), medications to be continued as previously prescribed, recommendations to crush the medications, recommendations to change to alternative formulations (an immediate-release dosage form instead of extended-release or an available liquid formulation instead of a pill formulation), and emphasizing the importance of monitoring blood pressure, blood sugar, lipid levels, and drug levels after surgery. Furthermore, the consultations highlight issues related to drug–drug interactions and interactions with specific medications and vitamins.

At the end of the appointment, a written summary is given to the surgeons and incorporated into hospital records. The inclusion of a clinical pharmacist as part of a multidisciplinary team for patients undergoing bariatric surgery is unique and not standard practice within Israeli bariatric surgical units.

The primary study outcomes included the rate of surgical complications and the rates of re-hospitalizations and mortality up to one year after surgery. In a secondary analysis, re-hospitalizations were compared in four different periods up to one year after surgery to differentiate between early and late complications.

### 2.1. Statistical Analysis

Demographic data, including age, sex, body mass index (BMI), and comorbidities, are presented as percentages, averages, and standard deviations. T-tests were used to examine statistical differences between continuous variables. Chi-square tests were used to examine relationships between categorical variables. A two-sided *p*-value of <0.05 was considered statistically significant.

The sample size required to demonstrate a 50% reduction in re-hospitalizations and mortality in the intervention group compared with the comparison group with 80% power, a 5% significance level, and a mortality/re-hospitalization rate of 20% in the comparison group was 24,950 (106 in the intervention group and 24,844 in the comparison group). The ratio between the comparison group and the intervention group was 1:235.

### 2.2. Approvals

The study was approved by the Helsinki Committee of Herzliya Medical Center and the Data Utilization Committee of CHS. Informed consent was not required due to the retrospective study design.

## 3. Results

During the study period, 210 bariatric patients received dedicated drug counseling from a clinical pharmacist at Herzliya Medical Center. Thirty-four patients were excluded from the study cohort since they did not belong to CHS, and another 11 patients were excluded because, despite being candidates for bariatric surgery, they did not receive operations. A final total of 165 CHS members were included in the intervention cohort. Of these, 49.7% received pharmaceutical advice before surgery, 41.2% received the consultation during hospitalization, and 9.1% received the consultation after surgery.

The comparison cohort included 38,854 bariatric patients who were reported to have underlying chronic diseases, which constituted about 70% of the general patient population reported in the bariatric registrar in those years. When comparing the intervention cohort and the comparison population, it was found that the intervention population tended to be older, had a higher proportion of males than females, and had higher rates of underlying diseases (Table 1).

Re-hospitalizations up to one year after surgery occurred in 10.9% of the intervention cohort versus 19.5% in the comparison group (*p* = 0.005). There were no deaths in the intervention group, and there were 62 deaths (0.16%) in the comparison group. Regarding surgical complications, there were no statistically significant differences between the intervention group and the comparison group—2.7% versus 3.9%, respectively (*p* = 0.462). The comparison of the primary outcomes between the study cohorts is presented in Table 2.

The results of the secondary analysis, regarding the proportion of re-hospitalizations at different time intervals after surgery, are presented in Table 3. During the timeframe of 30 days after bariatric surgery, re-hospitalizations in the intervention group were significantly lower than those in the control group—1.8% vs. 5.3%, respectively (*p* = 0.046). The rate of re-hospitalizations in the other time periods of 31–90, 91–180, and 181–365 days after surgery were 2.4% vs. 4.1% (*p* = 0.278), 3.6% vs. 4.8% (*p* = 0.476) and 7.3% vs. 9.9% (*p* = 0.256) in the intervention vs. comparison cohorts, respectively.

The Israeli General Bariatric Registry publishes annual data on the general patient population undergoing bariatric surgery. The data on re-hospitalization is reported up to 3 months after surgery. In the year 2017, among 7960 bariatric patients, 434 (5.5%) and 235 (2.9%) were re-hospitalized in the timeframes of 0–30 and 31–90 days after surgery, respectively. In the year 2018, among 7559 bariatric patients, 360 (4.7%) and 207 (2.7%) were re-hospitalized in the timeframe of 0–30 and 31–90 days after surgery, respectively.

Similar results were also observed when patients in the intervention group were compared with the general patient population undergoing bariatric surgery. During the time frame of 30 days after bariatric surgery, re-hospitalizations in the intervention group were significantly lower than those in the control group (*p* = 0.023). It is important to note that these results reinforce the findings we obtained in our study because the general bariatric population includes younger patients and those without comorbidities. The results are presented in Table 4.

## 4. Discussion

In this study, the provision of dedicated drug counseling to bariatric patients was found to be associated with a decrease in the rate of re-hospitalizations after surgery. In addition, in the intervention group, there were no deaths up to one year after surgery, even though the study patients were more complex both in terms of average age and background comorbidities.

Re-hospitalizations after surgery are a significant burden on healthcare resources and are a major inefficiency in the healthcare system. The total cost in the USA alone is estimated at $2.5 billion. In addition to increased costs, re-hospitalizations are associated with poorer surgical patient outcomes [21]. In this cohort study, a reduction in re-hospitalizations was observed in the one-month period after bariatric surgery and up to one year after surgery. According to the study results, if 38,854 patients received a clinical pharmacist consultation, we would expect 4238 patients to be hospitalized during the study period. This means that the number of patients who would have needed re-hospitalization was reduced by 3348 (8.6%). Depending on the number of re-hospitalizations, the length of stay, and the cost of in-patient hospitalization, the healthcare system is able to save substantial costs every year.

The clinical pharmacist’s consultation included a comprehensive assessment of the patient’s medications and medical records in order to match the changes in the digestive system that the patient was expected to undergo because of the bariatric surgery and the other effects of the surgery causing improvement in comorbidities, such as diabetes, hypertension, etc. The consultation was for patients but also was aimed at the healthcare providers treating the patients in the hospital and in the ambulatory care setting. Only a few related studies regarding the involvement of pharmacists in advising patients undergoing bariatric surgery are described in the literature.

Han et al. [22] reported that although there were no statistical differences in hospitalization periods between patients who received a pharmacist consultation before bariatric surgery and those who did not, the pharmacist had an effect on preventing potential side effects, and patients had a high level of satisfaction with the advice given to them.

Silverman et al. [23] reported on collaborations between surgeons and pharmacy staff in order to provide an efficient response to issues related to bariatric patients’ drug treatment. Van Prooven et al. [8] showed that the involvement of a clinical pharmacist in the adjustment of medications after bariatric surgery reduced prescriptions, doses, and formulations that were not adapted to bariatric patients.

In our current study, the usefulness of the pharmacist’s consultation regarding bariatric surgery was demonstrated, but in order to ensure that patient safety is maintained over a long period, a periodic evaluation of the drug treatment by a clinical pharmacist in the community is still required. Clinical pharmacists have been trained to identify problems with drug treatment and give recommendations that can reduce potential side effects, complications, and re-hospitalizations related to bariatric patients’ medicinal care [22]. Future comparative and randomized studies with longer follow-ups are needed to investigate the long-term effects of clinical pharmacy consultations on the morbidity and mortality of bariatric patients.

This study has several limitations, mainly due to the fact that the research was retrospective and not prospectively controlled. First, the data reported by the National Registrar were provided grouped in cohorts [20]. Individual data on each of the listed patients were not available for research. Therefore, it was not possible to adjust the results for all confounding variables that could have led to major biases in the results and erroneous conclusions about causal relationships. Second, all consultations were performed by one clinical pharmacist, and thus the results may not be repeated to the same extent in other studies. Third, the intervention group was statistically older and had more background diseases; despite this, there were fewer re-hospitalizations one month and one year after surgery. It is possible that if the groups were more similar, the results would have been even more significant. Although the clinical pharmacist who performed the consultations was the first one in Israel to have done so in the bariatric population, we cannot be entirely sure that the registry patients did not receive any consultations from other healthcare providers. However, the authors are very familiar with the healthcare community in this field, and the rate of patients that may have received consultations should not be statistically significant in comparison with the entire population of the registry, as can be seen in the results. As this is a retrospective study, patient satisfaction with the consultation and their understanding of the instructions given were not evaluated. Also, the long-term implementation of the recommendations was not shown.

In the past few years, more and more bariatric patients have received consultations with clinical pharmacists, although the rate of these consultations is thought to still be very small compared with the number of patients undergoing surgery and are organization-based. In addition to comorbidities related to the patient’s metabolic condition, consultations were also performed for critical medications such as for epilepsy, psychiatric conditions, cancer treatment and prevention, neurological conditions, and more.

Clinical pharmacists should be an integral part of the treating team and we believe that they should advise all patients as part of the pre-surgery bariatric committee that is mandated for patients undergoing surgery or at least those with chronic conditions. Long-term post-surgery follow-up to monitor the influence of the surgery on medications and nutritional deficiencies, and adjustment of drug therapy per clinical effect, is important for the medical success of patients as well. This can also be critical for choosing the type of surgery for risk management of patients’ chronic conditions so they will not worsen after surgery due to changes in drug absorption.

## 5. Conclusions

In this retrospective study, we demonstrated the benefit of a clinical pharmacist consultation regarding bariatric surgery for complex patients with risk factors for morbidity and mortality as a result of the surgery. Despite the relatively small number of patients in the intervention group, a sample of 165 patients was sufficient to obtain statistical significance.

To our knowledge, this is the first published study showing the long-term effects of a clinical pharmacy consultation in bariatric patients on hospitalization rates. Incorporating a clinical pharmacist into the work of the bariatric team seemed to increase the safety and efficacy of bariatric treatment. Additional prospective studies in larger intervention groups are required to confirm these findings and target counseling to special-risk populations. Our encouraging outcomes should hopefully stimulate more studies to show the invaluable role of specialized clinical pharmacists.

## Figures and Tables

**Table 1:** Baseline characteristics of the intervention and comparison cohorts.

	Intervention Cohort (N = 165)	Comparison Cohort (N = 38,854)	*p*-Value
*Demographic variables*			
Age, mean (SD)	52.9 (11.4)	42.7 (12.4)	<0.001
Sex—Male	92 (55.8%)	12,349 (31.8%)	<0.001
*Clinical risk factors*			
*BMI, mean kg/m^2^*, (SD)	41.3 (4.9)	41.8 (5.4)	0.235
Diabetes Mellitus	107 (64.8%)	11,142 (28.7%)	<0.001
Hypertension	112 (67.9%)	13,195 (34.0%)	<0.001
*Hyperlipidemia*	122 (73.9%)	11,454 (29.5%)	<0.001
*Depression/anxiety*	26 (15.8%)	3255 (8.4%)	<0.001
*Asthma*	7 (4.2%)	2744 (7.1%)	0.158
*COPD*	10 (6.1%)	793 (2.0%)	<0.001
*Cardiovascular disease*	40 (24.2%)	1640 (4.2%)	<0.001
*History of stroke*	4 (2.4%)	386 (1.0%)	0.065
*Sleep apnea*	43 (26.1%)	6481 (16.7%)	0.001
*Bariatric procedure*			
*Ring (band)*	*2* (1.2%)	1596 (4.1%)	
*Sleeve*	*111* (67.3%)	22,413 (57.7%)	
*Gastric bypass*	*20* (12.1%)	4635 (11.9%)	
*One anastomosis gastric bypass*	*32* (19.4%)	10,210 (26.3%)	

BMI, body mass index; COPD, chronic obstructive pulmonary disease.

**Table 2:** Primary outcomes in the intervention and comparison cohorts.

Outcome	Intervention Cohort (N = 165)	Comparison Cohort (N = 38,854)	*p*-Value
Re-hospitalization	18 (10.9%)	7586 (19.5%)	0.005
Death	0 (0%)	62 (0.16%)	NA
Surgical complications *	4 (2.7%)	1524 (3.9%)	0.462

* A total of 146 bariatric patients with documented medical hospitalization records were included. These patients received clinical pharmacist consultations either before surgery or during their hospitalization. NA, not applicable.

**Table 3:** Secondary outcome: re-hospitalizations in different time periods in the intervention vs. comparison cohorts. Patients were included once in each timeframe but could appear in several timeframes.

Time Period (Days after Surgery)	Intervention Cohort (N = 165)	Comparison Cohort (N = 38,854)	*p*-Value
0–30	3 (1.8%)	2062 (5.3%)	0.046
31–90	4 (2.4%)	1596 (4.1%)	0.278
91–180	6 (3.6%)	1876 (4.8%)	0.476
181–365	12 (7.3%)	3857 (9.9%)	0.256

**Table 4:** Re-hospitalizations in different time periods in the intervention group (2013–2019) vs. the general bariatric surgery population (2017–2018). Patients were included once in each timeframe but could appear in several timeframes.

Time Period (Days after Surgery)	Intervention Cohort 2013–2019 (N = 165)	Comparison 2017 (N = 7960)	Comparison 2018 (N = 7559)	*p*-Value
0–30	3 (1.8%)	434 (5.5%)	360 (4.7%)	0.023
31–90	4 (2.4%)	235 (2.9%)	207 (2.7%)	0.688

## Data Availability

The data presented in this study are available upon reasonable request from the corresponding authors.
